# Selective Spectrophotometric and Spectrofluorometric Methods for the Determination of Amantadine Hydrochloride in Capsules and Plasma via Derivatization with 1,2-Naphthoquinone-4-sulphonate

**DOI:** 10.1155/2009/810104

**Published:** 2009-01-25

**Authors:** Ashraf M. Mahmoud, Nasr Y. Khalil, Ibrahim A. Darwish, Tarek Aboul-Fadl

**Affiliations:** Department of Pharmaceutical Chemistry, College of Pharmacy, King Saud University, P.O. Box 2457, Riyadh 11451, Saudi Arabia

## Abstract

New selective and sensitive spectrophotometric and spectrofluorometric methods have been developed and validated for the determination of amantadine hydrochloride (AMD) in capsules and plasma. The methods were based on the condensation of AMD with 1,2-naphthoquinone-4-sulphonate (NQS) in an alkaline medium to form an orange-colored product. The spectrophotometric method involved the measurement of the colored product at 460  nm. The spectrofluorometric method involved the reduction of the product with potassium borohydride, and the subsequent measurement of the formed fluorescent reduced AMD-NQS product at 382  nm after excitation at 293  nm. The variables that affected the reaction were carefully studied and optimized. Under the optimum conditions, linear relationships with good correlation coefficients (0.9972–0.9974) and low LOD (1.39 and 0.013 *μ*g mL^−1^) were obtained in the ranges of 5–80 and 0.05–10 *μ*g mL^−1^ for the spectrophotometric and spectrofluorometric methods, respectively. The precisions of the methods were satisfactory; RSD ≤2.04%. Both methods were successfully applied to the determination of AMD in capsules. As its higher sensitivity, the spectrofluorometric method was applied to the determination of AMD in plasma; the recovery was 96.3–101.2 ± 0.57–4.2%. The results obtained by the proposed methods were comparable with those obtained by the official method

## 1. Introduction

**Scheme 1:**
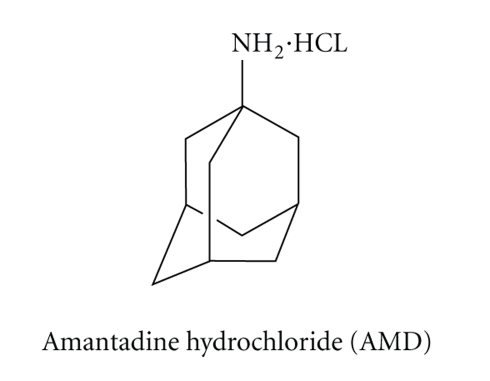


Amantadine hydrochloride (AMD), Scheme 1, is an antiviral agent used against infection with influenza type A virus and to ameliorate symptoms when administered during the early stages of infection as well as in the management of herpes zoster [1]. It has mild anti-Parkinsonism activity and thus it has been used in the management of Parkinsonism, mainly in the early disease stage and when the symptoms are mild. AMD is usually given by mouth as the hydrochloride salt [2].

Spectrophotometry and spectrofluorometry are considered as the most convenient analytical techniques in pharmaceutical analysis because of their inherent simplicity and availability in most quality control and clinical laboratories [3–9]. However, AMD does not possess any chromophore or fluorophore in its molecule, which are the essential requirements for the direct analysis by either spectrophotometric or spectrofluorometric techniques. Therefore, derivatization of AMD was necessary for its determination by either of the two techniques. For spectrophotometric determination of AMD, it has been derivatized with different reagents. The involved derivatization reactions that have been published prior to 1983 have been reviewed by Kirschbaum [10]. The derivatizing reagents used thereafter included iodine [11], acetaldehyde/chloranil [11], *α*,*α*-diphenyl-*β*-picrylhydrazyl [12], bromocresol green [13], tetracyanoethylene [14], iron(III) [15], and cyclodextrin [16]. Few spectrofluorometric methods have been reported for the analysis of AMD. These methods were based on its oxidation with cerium(IV) [7] or its derivatization with 2,3-diphenylquinolizinium bromide [17], fluorescamine [18], and 9-isothiocynatoacridine [19]. As well, many derivatization techniques coupled with chromatography have been established for the determination of AMD in the dosage forms and biological matrices: TLC [20], HPLC [21–23], GC [24], and capillary electrophoresis [25]. 1,2-Naphthoquinone-4-sulphonate (NQS) has been used for the determination of many compounds [26–28]. The reaction between NQS and AMD has not been investigated yet. Therefore, the present study was devoted to explore NQS as a derivatizing reagent in the development of selective and sensitive spectrophotometric and spectrofluorometric methods for the determination of AMD in capsules and plasma.

## 2. Experimental

### 2.1. Apparatus

UV-1601 PC (Shimadzu, Kyoto, Japan) ultraviolet-visible spectrophotometer with matched 1 cm quartz cells was used for all spectrophotometric measurements. Spectrofluorimeter, Kontron SFM 25 equipped with a 150 W xenon high-pressure lamp was used for measuring the fluorescence intensity. MLW type thermostatically controlled water bath (Memmert GmbH, Co. Schwa bach, Germany). Biofuge Pico centrifuge (Heraeus Instruments, Germany).

### 2.2. Chemicals and Materials

Amantadine hydrochloride (AMD; Sigma-Aldrich Chemie GmbH, Steinheim, Germany) was obtained and used as received, its purity was 100.02 ± 1.25%. 1,2-naphthoquinone-4-sulphonate; (NQS; El-Nasr Pharmaceutical Chemical Co., Abo-Zaabal, Egypt). Potassium borohydride (Sigma-Aldrich Chemie GmbH, Steinheim, Germany). Adamine capsules (Rameda Co. for Pharmaceutical Industries & Diagnostic Reagents, Cairo, Egypt) are labeled to contain 100 mg of AMD per capsule. Human plasma samples were collected from normal healthy volunteer at King Khaled University Hospital (Riyadh, Kingdom of Saudi Arabia), and they were stored at −20°C until analysis. All solvents and materials used throughout this study were of analytical grade. Double distilled water was obtained through WSC-85 water purification system (Hamilton Laboratory Glass Ltd., Ky, USA), and used throughout the work.

### 2.3. Preparation of Standard and Sample Solutions

#### 2.3.1. Amantadine Hydrochloride (AMD) Standard Solutions

An accurately weighed amount (100 mg) of AMD was quantitatively transferred into a 50 mL calibrated flask, dissolved in 30 mL distilled water, completed to volume with the same solvent to obtain a stock solution of 2 mg mL^−1^. This stock solution was further diluted with water to obtain working solutions in the ranges of 50–800 and 0.5–100 *μ*g  mL^−1^ for the spectrophotometric and spectrofluorometric methods, respectively.

#### 2.3.2. 1,2-Naphthoquinone-4-sulphonate (NQS) Derivatizing Reagent

Accurately weighed amount of NQS (150 mg) was transferred into a 25 mL calibrated flask, dissolved in 5 mL distilled water, completed to volume with water to obtain a solution of 0.6% (w/v). The solution was freshly prepared and protected from light during use.

#### 2.3.3. Capsules Sample Solution

Twenty capsules were carefully evacuated; their contents were weighed and finely powdered. An accurately weighed quantity of the capsule contents equivalent to 100 mg of AMD was transferred into a 100 mL calibrated flask, and dissolved in about 40 mL of distilled water. The contents of the flask were swirled, sonicated for 5 minutes, and then completed to volume with water. The contents were mixed well and filtered rejecting the first portion of the filtrate. The prepared solution was diluted quantitatively with distilled water to obtain a suitable concentration for the analysis.

#### 2.3.4. Spiked Plasma Samples

Aliquots of 1.0 mL of plasma were spiked with different concentration levels of AMD. The spiked plasma samples were treated with 0.1 mL of 70% perchloric acid and vortexed for 1 minute. The samples were centrifuged for 20 minutes at 13000 rpm. The supernatants were transferred into test tubes and neutralized with 1 M NaOH solution.

### 2.4. General Recommended Procedure

#### 2.4.1. Spectrophotometric Method

One milliliter of the standard or sample solution (50–800 *μ*g mL^−1^) was transferred into a test tube. One milliliter of 0.01 M NaOH and 1 ml of NQS reagent (0.6%, w/v) were added. The contents of the tubes were heated in a water bath at 80 ± 5°C for 45 minutes and then cooled in ice water for 2 minutes. The contents of the test tubes were transferred quantitatively into a separating funnel containing anhydrous sodium sulphate and extracted with two portions (5 mL) of chloroform. The combined chloroformic extracts were transferred into 10 mL calibrated flasks and the solutions were completed to volume with chloroform if necessary. The absorbances of the resulting solutions were measured at 460 nm against reagent blanks treated similarly.

#### 2.4.2. Spectrofluorometric Method

One milliliter of the standard or sample solution (0.5–100 *μ*g mL^−1^) was transferred into a test tube. One milliliter of 0.01 M NaOH and 1 mL of NQS reagent (0.6%, w/v) were added. The contents of the test tubes were heated in a water bath at 80 ± 5°C for 45 minutes and then cooled in ice water for 2 minutes. The contents of the test tubes were transferred quantitatively into a separating funnel and extracted with two portions (5 mL) of chloroform. The combined chloroformic extracts were evaporated under stream of air and the residues were reconstituted in 2 mL of methanol and quantitatively transferred into 10 mL calibrated flask. A one milliliter of KBH_4_ solution (0.03%, w/v in methanol) was added and the reaction was allowed to proceed for 5 minutes at room temperature (25 ± 5°C). The solution was diluted to volume with 0.025 M ethanolic HCl and the fluorescence intensity of the resulting solution was measured at 382 nm after excitation at 293 nm against reagent blanks treated similarly.

### 2.5. Determination of the Molar Ratio of the Reaction

The Job's method of continuous variation [29] was employed. Master equimolar (2.5 × 10^−2^   M) aqueous solutions of AMD and NQS were prepared. Series of 5 mL portions of the master solutions of AMD and NQS were made up comprising different complementary proportions (0 : 10, 1:9,…, 9 : 1, 10 : 0, inclusive) in test tubes. One milliliter of 0.01 M NaOH was added to each tube, and the tubes were further manipulated as described under the general recommended procedure for the spectrophotometric method.

## 3. Results and Discussion

### 3.1. Strategy for Assays Development, Involved Reaction, and Spectral Characteristics

**Figure 1:**
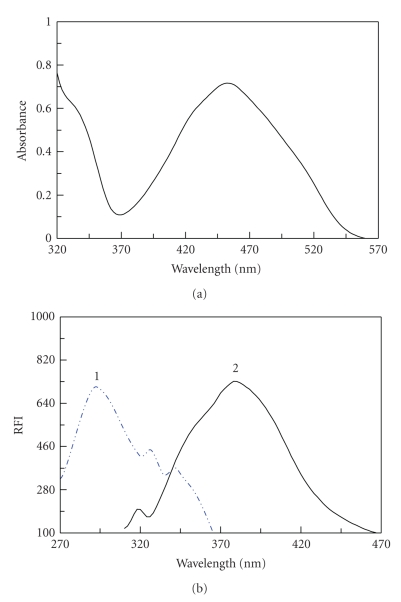
Absorption, excitation, and emission spectra on the reaction products of AMD with NQS (0.06%, w/v). (a) Absorption spectrum of the reaction product of AMD (65 *μ*g mL^−1^) with NQS after extraction with chloroform. (b) (1) Excitation and (2) emission spectra of the reduced reaction product. RFI is the relative fluorescence intensity.

**Scheme 2:**
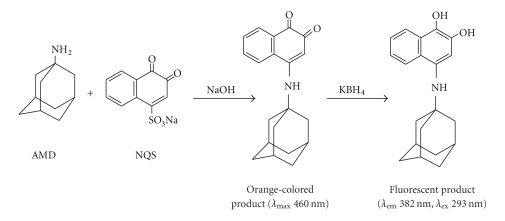
Scheme for the reaction pathway of amantadine hydrochloride (AMD) with 1,2-naphthoquinone-4-sulphonate (NQS).

Because of the absence of any chromophoric group in the AMD molecule, it has no absorption in the ultraviolet-visible region above 200 nm, and it has no native fluorescence as well. Therefore, direct spectrophotometric and fluorimetric determination of AMD were not possible. Therefore, derivatization of AMD was attempted in the present study for the development of both spectrophotometric and spectrofluorometric methods for its determination. NQS has been used as chromogenic and fluorogenic reagent for primary and secondary amines [26–28, 30], however, its reaction with AMD has not been investigated yet. Therefore, the present study was devoted to explore NQS as a derivatizing reagent in the development of spectrophotometric and spectrofluorometric methods for the determination of AMD in capsules and plasma. Our preliminary experiments in investigating the reaction between AMD and NQS revealed that NQS-AMD product is orange colored exhibiting a maximum absorption at 460 nm and it is insoluble in water, but soluble in organic solvents. Since the present work was directed to involve plasma samples, the interference of endogenous amines was a major concern. It is well known that NQS reacts with the endogenous amines (e.g., amino acids) and yields water-soluble products [30]. For this reason, an extraction step was necessary for the development of selective methods for the determination of AMD in the presence of the endogenous amines. As well, the reduced AMD-NQS derivative was found to be fluorescent and exhibited one emission maximum at 382 nm and three excitation maxima at 293, 325, and 344 nm. The highest fluorescence intensity was obtained after excitation 293 nm, thus the excitation in the present study was performed at this wavelength. Scheme 2 shows the reaction pathway between AMD and NQS, and Figure 1 shows the absorption, excitation, and emission spectra of the reaction product. The following sections describe the optimization of the assay variables and validation for the performance of both spectrophotometric and spectrofluorometric methods.

### 3.2. Method Development

#### 3.2.1. Optimization of Derivatization Reaction and Spectrophotometric Procedure

**Figure 2:**
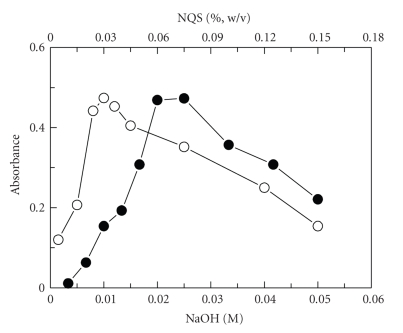
Effect of NaOH (∘) and NQS(•) concentrations on the reaction of AMD (45 *μ*g mL^−1^) with NQS.

**Figure 3:**
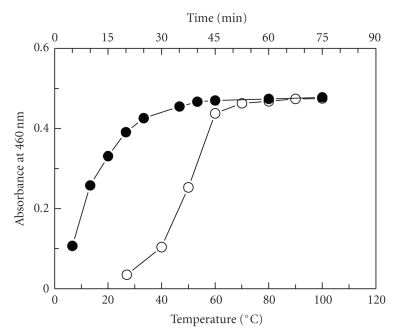
Effect of heating temperature (∘) and time (•) on the reaction of AMD (45 *μ*g mL^−1^) with NQS.

The factors affecting the derivatization reaction (the concentrations of NQS and NaOH, reaction time, temperature, diluting solvent, and the extracting solvent) were investigated by altering each variable in a turn while keeping the others constant. The studying of NQS concentrations revealed that the reaction was dependent on NQS reagent (Figure 2). The highest absorption intensity was attained when the concentration of NQS was 0.06–0.075% (w/v) in the final solution; further experiments were carried out at NQS concentration of 0.06% (w/v). The results of investigating the effect of NaOH concentration on the reaction revealed that the optimum NaOH concentration was 0.01 M (Figure 2). The effect of temperature on the derivatization reaction was studied by carrying out the reaction at different temperatures (25–100°C) and the maximum readings were obtained at 70–100°C (Figure 3). For more precise readings, further experiments were carried out at 80 ± 5°C. The effect of heating time on the formation of the reaction product was investigated by carrying out the reaction at different times. The maximum absorbance intensity was attained after 40 minutes, and longer reaction time did not affect the absorbance intensity (Figure 3). For more precise results, further experiments were carried out at 45 minutes.

It was found that the colored AMD-NQS product is insoluble in the aqueous reaction medium. For measurements, the reaction product might be either dissolved in a miscible organic solvent of lower polarity than water or extracted with an immiscible extractive solvent. Different solvents were tested for dilution; methanol, ethanol, acetonitrile, dimethylsulphoxide, isopropanol, 1,4-dioxane, and acetone. The highest readings were obtained when dioxane was used for dilution (data not shown). As well, different nonmiscible solvents were tested for the extraction of the AMD-NQS product: carbon tetrachloride, chloroform, dichloromethane, ethyl acetate, toluene, and benzene. The highest readings were obtained when chloroform was used for extraction (Table 1). The results revealed that the extractive procedure is more sensitive (1.5 fold) than the nonextractive procedure. This was attributed to the effective decrease in the blank readings and consequently enhanced the sensitivity of the assay.

#### 3.2.2. Optimization of Spectrofluorometric Procedure

**Figure 4:**
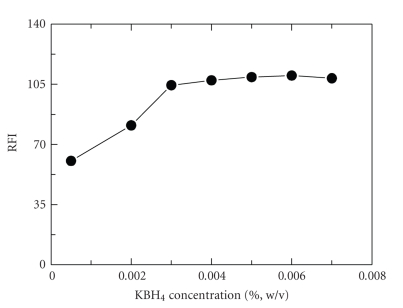
Effect of KBH_4_ concentration on the fluorescence intensity of the reaction product of AMD (1 *μ*g mL^−1^) with NQS (0.06%, w/v).

**Figure 5:**
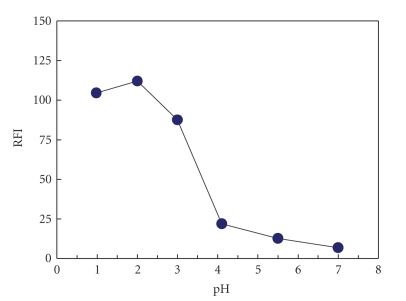
Effect of pH on the fluorescence intensity of the reduced reaction product of AMD-NQS. RFI is the relative fluorescence intensity.

For developing the spectrofluorometric procedure, a reduction step for AMD-NQS product was necessary. However, the reduced NQS reagent itself is also fluorescent and it had the same excitation and emission maxima of the AMD-NQS product, therefore, a selective extraction step for the AMD-NQS product from the remaining NQS reagent was necessary before carrying out the reduction step. Furthermore, the extraction step is essential to provide the required selectivity for analyzing the plasma samples as the NQS products with endogenous amines were water soluble. Based on the reported efficiency [30], potassium borohydride as a reducing reagent was selected for NQS-derivatives. In order to investigate the effect of potassium borohydride concentration on the reduction, the reaction was performed using varying concentrations (0.0005–0.01%, w/v). The highest fluorescence intensity was obtained when the concentration was 0.003% in the final solution (1 mL of 0.03%, w/v). Concentrations more than 0.003% did not affect the fluorescence intensity (Figure 4). The effect of pH on the fluorescence intensity was also studied and the results showed that the highest fluorescence intensity was obtained at pH 2.0 (Figure 5). This pH could be attained by diluting the reaction mixture with 0.025 M ethanolic HCl solution.

### 3.3. Stoichiometry of Derivatization Reaction

Under the optimum conditions, the stoichiometry of the reaction between AMD and NQS was investigated by Job's method [29] and was found to be 1:1 because AMD molecule contains only one center (primary amino group) available for this condensation reaction. Based on this ratio, the reaction pathway was postulated to be proceeded as shown in Scheme 2.

### 3.4. Method Validation

#### 3.4.1. Linearity, Limits of Detection and Quantitation

In the proposed methods, linear plots (*n* = 6) with good correlation coefficients (0.9974 and 0.9972) were obtained in the concentration ranges of 5–80 *μ*g mL^−1^ for and 0.05–10 *μ*g mL^−1^ for the spectrophotometric and the spectrofluorometric methods, respectively (Table 2). The limits of detection (LOD) and quantitation (LOQ) were determined [31] using the formula LOD or LOQ = *κ*SDa/b, where *κ *= 3.3 for LOD and 10 for LOQ, SDa is the standard deviation of the intercept, and b is the slope. The LOD values were 1.39 and 0.013 *μ*g mL^−1^ for the spectrophotometric and spectrofluorometric methods, respectively (Table 2).

#### 3.4.2. Precision and Accuracy

The precision of the proposed methods was determined by replicate analysis of five separate sample solutions at three concentration levels of AMD. The relative standard deviations (RSDs) were 0.83–0.96 and 0.46–1.01% for the spectrophotometric and spectrofluorometric methods, respectively (Table 3), indicating the good reproducibility of the proposed methods. Furthermore, the inter- and intra-assay precisions of the proposed spectrofluorometric method were determined from the recovery studies of spiked human plasma samples. The RSD values of the recovery were 0.57–2.04 and 0.72–4.20% for the intra- and inter-assay determinations, respectively (Table 4). The accuracy of the proposed methods was evaluated by standard addition method. The obtained recovery values were 98.8–100.2 ± 1.04–1.54% indicating the high accuracy of the proposed methods. Moreover, the accuracy of the proposed spectrofluorometric method was evaluated by the recovery studies of spiked human plasma samples. The obtained recovery values were 96.3–101.2 ± 0.57–4.2% (Table 4). These recovery results of the spiked human plasma indicate the suitability of the proposed spectrofluorometric method for the analysis of AMD in human plasma.

#### 3.4.3. Interference Studies

The results of the interferences study showed that no interferences were found from any of the excipients studied; lactose, sucrose, starch, talc, gum acacia, glucose, and magnesium stearate; the recovery of AMD was 98.15–100.72%. This indicated the absence of interferences from these excipients. Moreover, the interferences from the amino acids with the assay procedures were also studied using glycine as an example for the amino acids. The results of this study revealed that the amino acids could interfere with the spectrophotometric procedures. However, there is no any interference coming from the amino acids after an extraction step for the derivatized AMD product because the derivatized amino acids products are water soluble. Therefore, the extraction step increased both the sensitivity and selectivity by removing the interferences caused by both amino acids and proteins in the plasma samples.

#### 3.4.4. Robustness and Ruggedness

Robustness was examined by evaluating the influence of small variation of method variables, including concentration of analytical reagents and reaction time on the performance of the proposed methods. In these experiments, one parameter was changed whereas the others were kept unchanged, and the recovery percentage was calculated each time. It was found that small variation of method variables did not significantly affect the procedures. This provided an indication for the reliability of the proposed method during its routine application for the analysis of AMD. Ruggedness was also tested by applying the proposed methods to the assay of AMD using the same operational conditions but using two different instruments at two different laboratories and different elapsed time. Results obtained from lab-to-lab and day-to-day variations were found to be reproducible, the full range of recovery values was 98.4–101.3% and the RSD was 0.73 and 1.06% for the spectrophotometric and spectrofluorometric methods, respectively.

### 3.5. Application of the Proposed Method to Analysis of AMD in Capsules

It is evident from the above-mentioned results that the proposed methods gave satisfactory results with AMD in bulk. Thus, its capsules were subjected to the analysis of their contents from the active ingredient by the proposed methods and the official (nonaqueous titration) method [32]. The capsule contents, as percentage, were 98.70 ± 1.79 and 98.91 ± 1.93% for the spectrophotometric and spectrofluorometric methods, respectively (Table 5). These results were compared with those obtained from the official method by statistical analysis with respect to the accuracy (*t*-test) and precision (*F*-test). No significant differences were found between the calculated and theoretical values of *t*- and *F*-tests at 95% confidence limit proving similar accuracy and precision in the analysis of AMD in its dosage form.

## 4. Conclusions

The present study described the use of NQS reagent for the development of selective, sensitive, and accurate spectrophotometric and spectrofluorometric methods for the determination of AMD in bulk, capsules, and plasma. The described methods are superior to the previously reported spectrophotometric or spectrofluorometric methods for analysis of AMD in terms of their selectivity and sensitivity. The linear ranges of the proposed spectrophotometric and spectrofluorometric methods were 5–80 and 0.05–10 *μ*g/mL, respectively, which are much less than some reported methods 100–1300, 15–90, 25–75, 94–940 *μ*g/mL [11, 14, 15] which were based on either the nonselective oxidation or charge-transfer complex formation of AMD base. Although the sensitivity of the proposed spectrofluorimetric method is comparable to that described by Darwish et al. [7], which was based on the nonselective oxidation of AMD with cerric sulphate, however, our proposed methods are more selective. Also, The proposed methods involved spectrophotometric and spectrofluorometric measurements with comparable analytical performance devoid from any potential interference. This gives the advantage of flexibility in performing the analysis on any available instrument. Furthermore, all the analytical reagents are inexpensive, have excellent shelf life, and are available in any analytical laboratory. Therefore, these methods can be recommended for the routine analysis of AMD in quality control and clinical laboratories.

## Figures and Tables

**Table 1:** Effect of diluting and extracting solvents on the intensity of the reaction product of AMD with NQS (0.06%, w/v). Values for all solvents are mean of three determinations; the RSDs for the readings were <3.

Spectrophotometric method		Spectrofluorometric method	
Extracting solvent	Absorbance	Diluting solvent	Fluorescence intensity
Carbon tetrachloride	0.487	Methanol	65.66
Chloroform	0.718	Ethanol	78.38
Dichloromethane	0.616	Isopropanol	63.02
Ethyl-acetate	0.362	Acetone	2.00
Toluene	0.267	Acetonitrile	16.18
Benzene	0.239	Dimethylformamide	3.94
		1,4-Dioxane	76.11

**Table 2:** Quantitative parameters and statistical data for determination of amantadine hydrochloride by the proposed spectrophotometric and spectrofluorometric methods.

Parameter	Spectrophotometric method	Spectrofluorometric method
Range (*μ*g mL^−1^)	5.00–80.0	0.05–10.0
Intercept (a)	0.0759 ± 0.0041	−2.457 ± 0.332
Slope (b)	0.00974 ± 0.00035	81.969 ± 2.154
Correlation coefficient (r)	0.9974	0.9972
*ε* × 10^3^ (L mol^−1^ cm^−1^)	2.058	—
LOD (*μ*g mL^−1^)	1.39	0.013
LOQ (*μ*g mL^−1^)	4.21	0.041

**Table 3:** The precision of the proposed methods at three concentration levels of AMD.

Method	Nominal concentration (*μ*g mL^−1^)	RSD%^(a)^
Spectrophotometric	8.0	0.96
	40.0	0.83
	60.0	0.87
Spectrofluorometric	0.1	1.01
	4.0	0.58
	8.0	0.46

^(a)^Values are mean of five determinations.

**Table 4:** Recovery studies for the proposed spectrofluorometric method for the determination of AMD in spiked human plasma.

Spiked concentration (*μ*g mL^−1^)	Recovery (% ± RSD)^(a)^	
	Intra-assay	Inter-assay
0.05	98.2 ± 2.04	96.3 ± 4.20
0.10	99.3 ± 1.01	101.1 ± 2.0
0.20	100.5 ± 0.99	99.1 ± 1.50
0.40	100.9 ± 0.74	98.5 ± 1.52
0.80	100.1 ± 0.75	101.2 ± 1.44
1.60	98.6 ± 0.82	100.3 ± 1.12
3.20	98.9 ± 0.57	99.7 ± 0.72

^(a)^Values are mean of three and five determinations for intra- and inter-assay, respectively.

**Table 5:** Analysis of AMD in capsules by the proposed and official methods.

Method	Recovery (% ± SD)^(a)^	*t*-value^(b)^	*F*-value^(b)^
Spectrophotometric	98.70 ± 1.79	0.42	1.64
Spectrofluorometric	98.91 ± 1.91	0.19	1.85
Official HPLC^(c)^	99.13 ± 1.41	—	—

^(a)^Values are mean of five determinations.

^(b)^Theoretical values for *t* and *F* at 95% confidence limit and *n* = 5 were 2.31 and 6.39, respectively.

^(c)^Reference [32].

## References

[1] Prud'homme IT, Zoueva O, Weber JM (1997). Amantadine susceptibility in influenza A virus isolates: determination methods and lack of resistance in a Canadian sample, 1991–94. *Clinical and Diagnostic Virology*.

[2] Martindale (2002). *The Complete Drug Reference*.

[3] Darwish IA, Refaat IH, Askal HF, Marzouq MA (2006). Generic nonextractive spectrophotometric method for determination of 4-quinolone antibiotics by formation of ion-pair complexes with *β*-naphthol. *Journal of AOAC International*.

[4] Askal HF, Refaat IH, Darwish IA, Marzouq MA (2008). A selective spectrophotometric method for determination of rosoxacin antibiotic using sodium nitroprusside as a chromogenic reagent. *Spectrochimica Acta Part A*.

[5] Darwish IA, Khedr AS, Askal HF, Mohamed RM (2006). Application of inorganic oxidants to the spectrophotometric determination of ribavirin in bulk and capsules. *Journal of AOAC International*.

[6] Darwish IA, Hussein SA, Mahmoud AM, Hassan AI (2008). A sensitive spectrophotometric method for the determination of H 2-receptor antagonists by means of N-bromosuccinimide and p-aminophenol. *Acta Pharmaceutica*.

[7] Darwish IA, Khedr AS, Askal HF, Mahmoud RM (2005). Simple fluorimetric method for determination of certain antiviral drugs via their oxidation with cerium (IV). *Farmaco*.

[8] Darwish IA, Abdel-Wadood HM, Abdel-Latif N (2005). Validated spectrophotometric and fluorimetric methods for analysis of clozapine in tablets and urine. *Annali di Chimica*.

[9] Abdel-Wadood HM, Mohamed NA, Mahmoud AM (2008). Validated spectrofluorometric methods for determination of amlodipine besylate in tablets. *Spectrochimica Acta Part A*.

[10] Kirschbaum J (1983). *Analytical Profile of Drug Substances*.

[11] Darwish IA, Khedr AS, Askal HF, Mahmoud RM (2006). Simple and sensitive spectrophotometric methods for determination of amantadine hydrochloride. *Journal of Applied Spectroscopy*.

[12] Salman S, Bayrakdur N (1983). *Eczaclik Bull.*.

[13] Sultan M (2004). Spectrophotometric determination of amantadine in dosage forms. *Current Topics in Analytical Chemistry*.

[14] Rizk MS, Toubar SS, Sultan MA, Assaad SH (2003). Ultraviolet spectrophotometric determination of primary amine-containing drugs via their charge-transfer complexes with tetracyanoethylene. *Microchimica Acta*.

[15] Mustafa AA, Abdel-Fattah SA, Toubar SS, Sultan MA (2004). Spectrophotometric determination of acyclovir and amantadine hydrochloride through metals complexation. *Journal of Analytical Chemistry*.

[16] Kuwabara T, Nakajima H, Nanasawa M, Ueno A (1999). Color change indicators for molecules using methyl red-modified cyclodextrins. *Analytical Chemistry*.

[17] Martin MA, Del Castillo B (1991). 2,3-diphenylquinolizinium bromide as a fluorescent derivatization reagent for amines. *Analytica Chimica Acta*.

[18] De Silva JAF, Stronjny N (1975). Spectrofluorometric determination of pharmaceuticals containing aromatic or aliphatic primary amino groups as their fluorescamine (Fluram) derivatives. *Analytical Chemistry*.

[19] Sinsheimer JE, Hong DD, Stewart JT, Fink ML, Burckhalter JH (1971). Fluorescent analysis of primary aliphatic amines by reaction with 9-isothiocyanatoacridine. *Journal of Pharmaceutical Sciences*.

[20] Askal HF, Khedr AS, Darwish IA, Mahmoud RM (2008). Quantitative thin-layer chromatographic method for determination of amantadine hydrochloride. *International Journal of Biomedical Science*.

[21] Duh T-H, Wu H-L, Pan C-W, Kou H-S (2005). Fluorimetric liquid chromatographic analysis of amantadine in urine and pharmaceutical formulation. *Journal of Chromatography A*.

[22] Higashi Y, Fujii Y (2005). Simultaneous determination of the binding of amantadine and its analogues to synthetic melanin by liquid chromatography after precolumn derivatization with dansyl chloride. *Journal of Chromatographic Science*.

[23] Higashi Y, Nakamura S, Matsumura H, Fujii Y (2006). Simultaneous liquid chromatographic assay of amantadine and its four related compounds in phosphate-buffered saline using 4-fluoro-7-nitro-2,1,3-benzoxadiazole as a fluorescent derivatization reagent. *Biomedical Chromatography*.

[24] Leis HJ, Fauler G, Windischhofer W (2002). Quantitative analysis of memantine in human plasma by gas chromatography/negative ion chemical ionization/mass spectrometry. *Journal of Mass Spectrometry*.

[25] Reichová N, Pazourek J, Poláková P, Havel J (2002). Electrophoretic behavior of adamantane derivatives possessing antiviral activity and their determination by capillary zone electrophoresis with indirect detection. *Electrophoresis*.

[26] Gallo-Martinez L, Sevillano-Cabeza A, Campíns-Falcó P, Bosch-Reig F (1998). A new derivatization procedure for the determination of cephalexin with 1,2-naphthoquinone 4-sulphonate in pharmaceutical and urine samples using solid-phase extraction cartridges and UV-visible detection. *Analytica Chimica Acta*.

[27] Wang HY, Xu LX, Xiao Y, Han J (2004). Spectrophotometric determination of dapsone in pharmaceutical products using sodium 1,2-naphthoquinone-4-sulfonic as the chromogenic reagent. *Spectrochimica Acta Part A*.

[28] Darwish IA (2005). Kinetic spectrophotometric methods for determination of trimetazidine dihydrochloride. *Analytica Chimica Acta*.

[29] Job P (1964). *Advanced Physicochemical Experiments*.

[30] Pesez M, Bartos J (1974). *Colorimetric and Fluorimetric Analysis of Organic Compounds and Drugs*.

[31] ICH guideline Validation of Analytical Procedures: Text and Methodology.

[32] The United States Pharmacopeia 25

